# The life and death of the dominant follicle

**DOI:** 10.21451/1984-3143-AR2018-0030

**Published:** 2018-08-03

**Authors:** Christopher A Price, Anthony Estienne

**Affiliations:** Centre de Recherche en Reproduction et Fertilité, Faculty of Veterinary Medicine, University of Montreal, St-Hyacinthe, QC, Canada

**Keywords:** apoptosis, atresia, follicle, granulose.

## Abstract

Much work has been conducted over the years to determine the major factors that control follicle growth, including the role of FSH, LH and IGF1. These factors permit the dominant follicle to grow while subordinate follicles regress. The dominant follicle enters a phase of growth, and then that growth slows as the follicle reaches maximum size. The dominant follicle remains morphologically larger for a few days in the static phase, before starting to regress with the loss of functional dominance. Few studies have addressed the factors that determine follicle fate during the static phase. In this review, we summarize the differences in gene expression between growing and non-growing (static or early regressing) dominant follicles, highlighting areas that require further study. Potential factors that may help survival of the dominant follicle include IGF1, estradiol and BMP4/BMP7, and intrafollicular factors that likely initiate regression and apoptosis include FGF18 and AMH acting through FASLG. It is also very likely that the influence of microRNAs, especially miR-21, play a role in determining the fate of the dominant follicle.

## Introduction

The bovine dominant follicle, once established, continues to grow from about 9 to 15 mm diameter over the course of about 4 days, then enters a 4 to 5-day plateau or static phase with little further growth, after which it starts to regress concomitantly with the recruitment of a new follicle wave. The dynamics of dominant follicle growth and gonadotropin control of the establishment of follicular dominance in ruminants have been extensively reviewed (Ireland *et al*., 2000; Ginther, 2016; Shimizu, 2016; Webb *et al*., 2016). The growing dominant follicle is highly estrogenic and the granulosa cells proliferate as the follicle increases in diameter, initially under the influence of FSH. As plasma FSH concentrations decline, the continued growth of the follicle is supported by LH and IGF1 (Shimizu, 2016). As the follicle reaches the end of the growth phase, intrafollicular concentrations of estradiol decrease (Ireland and Roche, 1983; Badinga *et al*., 1992; Price *et al*., 1995) and the follicle enters the static phase.

It is well known that the dominant follicle is functionally as well as morphologically dominant, as it suppresses the development of smaller follicles; ablation of the dominant follicle allows immediate recruitment of a new follicle wave or can rescue the regressing subordinate follicle if performed early during the growth of the dominant follicle (Ko *et al*., 1991; Siddiqui *et al*., 2015). The static phase, despite its name, is a period of change for the dominant follicle and the fate of the follicle is decided during this time. Early static dominant follicles are estrogen-active and approximately half of static dominant follicles are morphologically healthy by light microscopy (Price *et al*., 1995; Irving-Rodgers *et al*., 2001), and they frequently respond to induced luteolysis by ovulating (Ali *et al*., 2001). In contrast, the late static dominant follicle is estrogen-inactive, mostly atretic and fails to ovulate after luteolysis (Ali *et al*., 2001; Irving-Rodgers *et al*., 2001). Therefore, the static phase is a plastic period of the dominant follicle lifespan during which the fate of the follicle is determined.

To determine the mechanisms of follicle growth and regression, many studies have been performed comparing growing dominant with regressing subordinate follicles of the same wave. Although these studies are of great value and have identified many characteristics of growth and regression, they do not address the 'static' phase of the dominant follicle lifespan. The purpose of this review is to summarize our understanding of the physiology of late growing, static and early regressing dominant follicles in cattle (*Bos taurus*), with reference to other species where appropriate, and to explore potential intrafollicular mechanisms that may determine follicle fate during the static phase. Several factors involved in early dominant follicle growth are described out of necessity when data for the late growing/static phase are unavailable.

## Gene expression patterns in late growing, static & early regressing dominant follicles

Numerous studies of the expression of candidate genes and of global transcriptomics have been performed comparing granulosa cells from dominant follicles and from subordinate follicles during and after selection in cattle (reviewed in Zielak-Steciwko and Evans, 2016), but less attention has been paid to the different phases of dominant follicle growth. An early series of studies measured steroidogenic enzyme and gonadotropin receptor mRNA levels by in-situ hybridization (reviewed in Bao and Garverick, 1998), and as follicle growth slowed in healthy dominant follicles (from day 4 to 6 after wave emergence), there were decreases in *CYP11A1* mRNA levels in theca and granulosa cells, and of *CYP17A1* and *LHCGR* in theca cells. These same studies demonstrated that atretic dominant follicles contained less *CYP19A1* and *LHCGR* mRNA in the granulosa cell layer compared to healthy dominant follicles of the same size. In a later study, a comparison between dominant follicles in the early and late growing phases, from day 2 to day 5.5 after wave emergence, demonstrated that as the dominant follicle reaches maximum diameter, granulosa *FSHR* mRNA levels decrease and *LHCGR* mRNA levels increase (Mihm *et al*., 2006), however this study included a number of smaller follicles that were no larger than the next subordinate follicle.

Other candidate gene studies have revealed that some fibroblast growth factors have been shown to differ between healthy and atretic dominant follicles; FGF18 is expressed in theca cells and mRNA levels are higher in atretic compared with healthy large follicles and in subordinate vs dominant follicles (Portela *et al*., 2010). FGF9 is predominantly expressed in granulosa cells and mRNA levels are higher in atretic compared with healthy large follicles (Schütz *et al*., 2016). In contrast, thecal *FGF2* and *FGF10* mRNA levels have been reported to be higher in healthy compared to atretic bovine follicles of abattoir origin (Berisha *et al*., 2004; Buratini *et al*., 2007).

Some members of the bone morphogenetic protein family also change with follicle health: Glister *et al*. demonstrated that granulosa cell *BMP2* mRNA levels decreased as follicle size increased (from 7 to 18 mm, abattoir ovaries; Glister *et al*., 2010) whereas Selvaraju at al. showed that BMP2 mRNA levels increase as dominant follicles progressed from pre- to mid- dominance (8 - 16 mm; timed collection) and remained high in static phase follicles (Selvaraju *et al*., 2013). Comparing estrogen-active and inactive large follicles, granulosa cell *BMP2* mRNA levels were higher in large atretic follicles compared to large healthy follicles (Glister *et al*., 2010). This latter study also showed that *BMP4, BMP6* and *BMP7* mRNA levels did not differ between healthy and unhealthy large follicles.

Cocaine- and amphetamine-regulated transcript (CARTPT) mRNA levels are very low in granulosa cells of dominant follicles in cattle compared with pre- selection follicles and do not change during dominant follicle growth (Lv et al., 2009). Unfortunately, data are not available for these genes in growing, static and early regressing dominant follicles.

Two studies have investigated the follicular transcriptome during the late growing/static phase of the dominant follicle lifespan. In one study, the static phase was mimicked in cows by stimulating with FSH followed by a 'coasting' period; in this model, the abundance of multiple mRNA species changed as the follicle coasts, with most changes reflecting an increase in genes encoding anti-proliferative and pro-apoptotic proteins as the static phase progresses (Nivet *et al*., 2013). In a study of follicles >9 mm diameter grouped as growing, static and regressing by flow cytometry, microarray analysis identified a number of genes that were differentially expressed between the three groups, suggesting that the follicles undergo distinct changes rather than a gradual slide from healthy to atretic (Girard *et al*., 2015). In this latter study, *BMP4* mRNA levels were not different between growing, static and regressing dominant follicles, in agreement with Glister *et al*. (2010).

MicroRNAs have also been the target of profiling during follicle growth. Using large bovine follicles of abattoir origin, 57 miRNA were differently expressed in estrogen-active compared to estrogen- inactive follicles (Sontakke *et al*., 2014). In a study comparing the dominant and subordinate follicles on day 3 and day 7 of the cycle, Salilew-Wondim and colleagues found few (16) differentially expressed miRNA between dominant and subordinate follicles on day 3, and a larger number (108) differentially expressed on day 7 (Salilew- Wondim *et al*., 2014). A direct comparison of dominant follicles on day 3 (growing) and day 7 (static or regressing) identified 131 differentially expressed miRNA in granulosa cells (Salilew-Wondim *et al*., 2014). The number of miRNA that were differentially expressed in both these datasets is small (Table 1), which might be a reflection of the different biological models used.

A study comparing preovulatory dominant follicles with subordinate follicles identified 34 miRNA enriched and 30 miRNA reduced in granulosa cells of preovulatory follicles compared to subordinate follicles; interestingly, PCR analysis indicated that selected miRNA differentially expressed in granulosa cells were also differentially expressed in theca cells (Gebremedhn *et al*., 2015). Preovulatory follicles would be expected to be different from non-ovulatory dominant follicles owing to the considerable increase in oestradiol levels and LH pulse frequency as well as a decrease in peripheral progesterone levels, so this particular comparison is not so relevant for the current discussion.

No global profiling has yet been reported for theca cells during this stage of follicle growth. A microarray study compared the theca cells of healthy and atretic follicles 3-5 mm diameter of abattoir origin, and concluded that most differentially expressed genes were related to inflammation and vascularization rather than apoptosis (Hatzirodos *et al*., 2014).

A glycoproteomic study was conducted with bovine granulosa and theca samples and atresia was associated with increased levels of certain sulphated chondroitin polysaccharides in granulosa cells and of sulphated heparan polysaccharides in theca cells (Hatzirodos *et al*., 2012). It is not known whether such changes occur during the growing-static-regressing phases of the dominant follicle.

**Table 1:** MicroRNA identified in both Sontakke et al. (2014) and Salilew-Wondim et al. (2014) as differentially expressed between growing and non-growing large follicles.

Upregulated in growing follicles	Upregulated in non-growing follicles
bta-miR-202	bta-miR-149-3p
bta-miR-31	bta-miR-21
bta-miR-873	bta-miR-150
bta-miR-652	bta-miR-204-3p
bta-miR-450b	bta-miR-409a
bta-miR-15b	bta-miR-142
bta-miR-424-p5	

## Prolonging the life of the dominant follicle

### FSH, IGF1 and estradiol

FSH is a major folliculogenic factor and administration of exogenous FSH induces the growth of multiple dominant follicles in a superovulatory setting; lower levels of FSH are also able to maintain the growth of 2 dominant follicles and/or delay regression of subordinate follicles (Adams *et al*., 1993; Mihm *et al*., 1997; Rivera and Fortune, 2001). This is likely to be caused by stimulation of the early growth phase of the dominant follicle which permits the selection of multiple dominant follicles. Once the dominant follicle is established it can remain viable in the face of low circulating FSH concentrations, and continue to grow past its normal lifespan if pulsatile LH secretion is increased (Stock and Fortune, 1993; Bigelow and Fortune, 1998). A regressing dominant follicle (as well as subordinate follicles) can also be rescued if FSH levels are increased (Ginther *et al*., 2016).

Intrafollicular estradiol is another major player involved in follicle health. It is well known that estradiol increases proliferation of granulosa cells of numerous species (Drummond and Findlay, 1999; Rosenfeld *et al*., 2001), enhances cell cycle progression from G1 to S phase (Quirk *et al*., 2006), and protects cells against FASLG- and FGF18-induced apoptosis (Quirk *et al*., 2006; Portela *et al*., 2015). It should be noted here that exogenously administered systemic estradiol induces atresia of the dominant follicle (Burke *et al*., 2005) by reducing LH pulse amplitude as well as FSH secretion (Price and Webb, 1988).

Although IGF1 is probably best known for its role in follicle deviation and the establishment of dominance, it also likely plays a role in supporting dominant follicle survival through regulation of IGF binding proteins (IGFBP) and hence IGF1 bioactivity (Mazerbourg and Monget, 2018). Large estrogen-active sheep and cattle follicles contain lower levels of IGFBPs - and thus higher IGF bioavailability - than do smaller growing or atretic follicles (Besnard *et al*., 1996; Roberts and Echternkamp, 2003), and addition of IGF1 to granulosa cells increased cell proliferation and estradiol secretion in a follicle-size specific manner (Monniaux and Pisselet, 1992; Gong *et al*., 1993; Spicer *et al*., 1993).

### The transforming growth factor-β (TGFβ) family

The TGFβ superfamily roles in ovarian function are critical and complex, and it seems that the disappearance of one of them or a disturbance of the equilibrium formed by these factors will strongly influence folliculogenesis and then ovulation. The role of TGFβ superfamily members in preantral follicle development and follicle selection has been reviewed (Knight and Glister, 2006).

Two BMPs, BMP4 and BMP7, are generally considered to be theca-derived proteins that act on granulosa cells. In cattle, both BMP4 and BMP7 mRNA are detected predominantly in theca cells (Fatehi *et al*., 2005; Glister *et al*., 2010), whereas in sheep neither BMP4 nor BMP7 mRNA were detected in follicles by in-situ hybridization (Juengel *et al*., 2006). Neither BMP4 nor BMP7 mRNA levels appear to be regulated by LH in bovine theca cells (Glister *et al*., 2011).

*In vitro* studies have shown that these BMPs affect granulosa cell steroidogenesis and proliferation. Theca-derived BMP4 and BMP7 increased granulosa proliferation and estrogen secretion, and inhibited progesterone synthesis in some studies with ruminants (Glister *et al*., 2004), but did not alter granulosa proliferation in another study (Yamashita *et al*., 2011). The effect of BMP7 on progesterone synthesis is due to a reduction of *STAR* mRNA levels (Yamashita *et al*., 2011). These BMPs may also promote follicle development/survival by increasing granulosa cell VEGF secretion and angiogenesis (Shimizu *et al*., 2012). Paradoxically, BMP4 and BMP7 potently inhibit androgen secretion from bovine theca cells (Glister *et al*., 2005) and as levels of neither appear to change with follicle health (Glister *et al*., 2010), the physiological role of these proteins remains to be established.

Levels of granulosa cell *BMP2* mRNA were lower in estrogen active dominant follicles compared with smaller growing follicles, and tended to increase in atretic follicles in cattle (Glister *et al*., 2010), and in sheep *BMP2* mRNA was only detected by in-situ hybridization in atretic follicles (Juengel *et al*., 2006). In contrast, *BMP2* mRNA levels were higher in large estrogen active dominant follicles compared with smaller growing follicles in water buffalo (Rajesh *et al*., 2018). Addition of recombinant BMP2 increased estradiol secretion but decreased progesterone secretion from ovine and bovine granulosa cells in vitro without altering cell proliferation (Souza *et al*., 2002; Juengel *et al*., 2006; Selvaraju *et al*., 2013).

Two other BMPs of interest are BMP15 and GDF9, which are expressed in the oocyte. GDF9 is critical for primary follicle growth and knock-out of *Gdf9* in the mouse results in arrest of folliculogenesis at the primary stage (Dong *et al*., 1996). In sheep, the role of GDF9 appears similar because in the case of a natural loss of function mutation of GDF9 in several breeds of ewes show abnormal folliculogenesis with arrest of follicle development at the primary stage (Nicol *et al*., 2009). Loss of *Bmp15* in mice results in reduced litter size owing to ovulation defects (Yan *et al*., 2001). In sheep there are several natural mutations that alter antral follicle growth, including *FecX^I^, FecX^R^* and *FecX^L^* , for which homozygous ewes are sterile with follicle arrest at the primary stage whereas ewes heterozygous for this same mutation have increased ovulation rate (Galloway *et al*., 2000; Bodin *et al*., 2007; Martinez-Royo *et al*., 2008). Another mutation in the BMP subfamily, called *FecB*, is in the coding sequence of the *BMPR1B* gene and induces a partial loss of function of this BMP receptor which leads to increased ovulation rate (Souza *et al*., 2001) and influences the proliferation and steroidogenesis of granulosa cells (Mulsant *et al*., 2001; Campbell *et al*., 2006). Recently, a mutation in a BMP signalling pathway termed 'Trio' has and been described in cattle, which results in increased *SMAD6* mRNA levels and, similar to the situation in sheep, in the growth and survival of two or more dominant follicles (Garcia-Guerra *et al*., 2018).

Both BMP15 and GDF9 affect granulosa cell proliferation and steroidogenesis, but in complex species-specific patterns. Recombinant BMP15 stimulated granulosa cell proliferation in ruminants (McNatty *et al*., 2005) and protects granulosa and cumulus cells against apoptosis in ruminants (Hussein *et al*., 2005). In sheep and cattle, BMP15 and GDF9 have been reported to inhibit FSH-induced progesterone synthesis by granulosa cells (McNatty *et al*., 2005; Fabre *et al*., 2006), although species of origin of the protein has been reported to alter its effect: ovine GDF9 inhibited progesterone secretion from sheep granulosa cells whereas mouse GDF9 increased progesterone secretion (McNatty *et al*., 2005). There is also a species difference in the amounts of BMP15/GDF9 secreted by the oocyte of polyovular vs monovular species, as sheep secrete both whereas rats secrete primarily GDF9 (Lin *et al*., 2012). BMP15 and GDF9 synergize, and this may be in the form of secreted heterodimers (cumulin) or secreted monomeric proteins that form dimers at the receptor of the target cell (Mottershead *et al*., 2015; Heath *et al*., 2017).

There is also evidence that GDF9 can alter theca cell function in pre-selection follicles, as it decreased proliferation and steroidogenesis of bovine theca cells from follicles <6 mm diamater, but had no effect on theca cells from follicles >8 mm diameter (Spicer *et al*., 2008).

## Induction of atresia in the dominant follicle

The fate of the dominant follicle is determined during the static phase of development, and the follicle can regress and become atretic 'passively' if the survival signals described above are reduced/absent. However, it is not clear what endocrine changes occur between the end of the growing period and the end of the static period. Alternatively, intrafollicular events may predispose a follicle toward atresia; the following section describes some potential pro-apoptotic factors that may play a role in determining the fate of the dominant follicle.

### Fas antigen and Fas ligand

Fas antigen is a transmembrane receptor which induces apoptosis when activated by the protein Fas ligand (FASLG). In cattle, granulosa cell *FAS* mRNA levels were not different between growing and atretic dominant follicles, but were significantly higher in the theca layer of atretic compared with healthy dominant follicles (Porter *et al*., 2000). *FASLG* mRNA levels are also higher in atretic vs healthy follicles in non- ruminants, and can be increased in ruminant granulosa and theca cells in vitro by treatments that increase apoptosis including serum withdrawal (Hu *et al*., 2001), FGF18 (Portela *et al*., 2015) and toxins (Guerrero-Netro *et al*., 2015, 2017). Alone, FASLG does not cause apoptosis in granulosa cell cultures with serum but requires the presence of IFN gamma - however, in serum- free culture, bovine GC are susceptible to FASL-induced apoptosis (Quirk *et al*., 2000), although this was prevented in the presence of IGF, FGF2 and EGF, but not FGF7, TGF, PDGF or gonadotropins (Quirk *et al*., 2000).

In rodents, Faslg induces granulosa cell death and decreased levels of inducible nitric oxide synthase (Nos2) mRNA levels, and this can be prevented by nitric oxide (Chen *et al*., 2005). In cattle, inhibition of endogenous NO production increased FASLG expression and granulosa cell apoptosis (Zamberlam *et al*., 2011). Estradiol stimulated *NOS2* mRNA levels in bovine granulosa cells (Zamberlam *et al*., 2011) and also attenuates FASLG-induced apoptosis (Quirk *et al*., 2006). It seems likely that FASLG is a mediator of apoptosis induced by various effectors.

### Fibroblast growth factors

**Figure 1:**
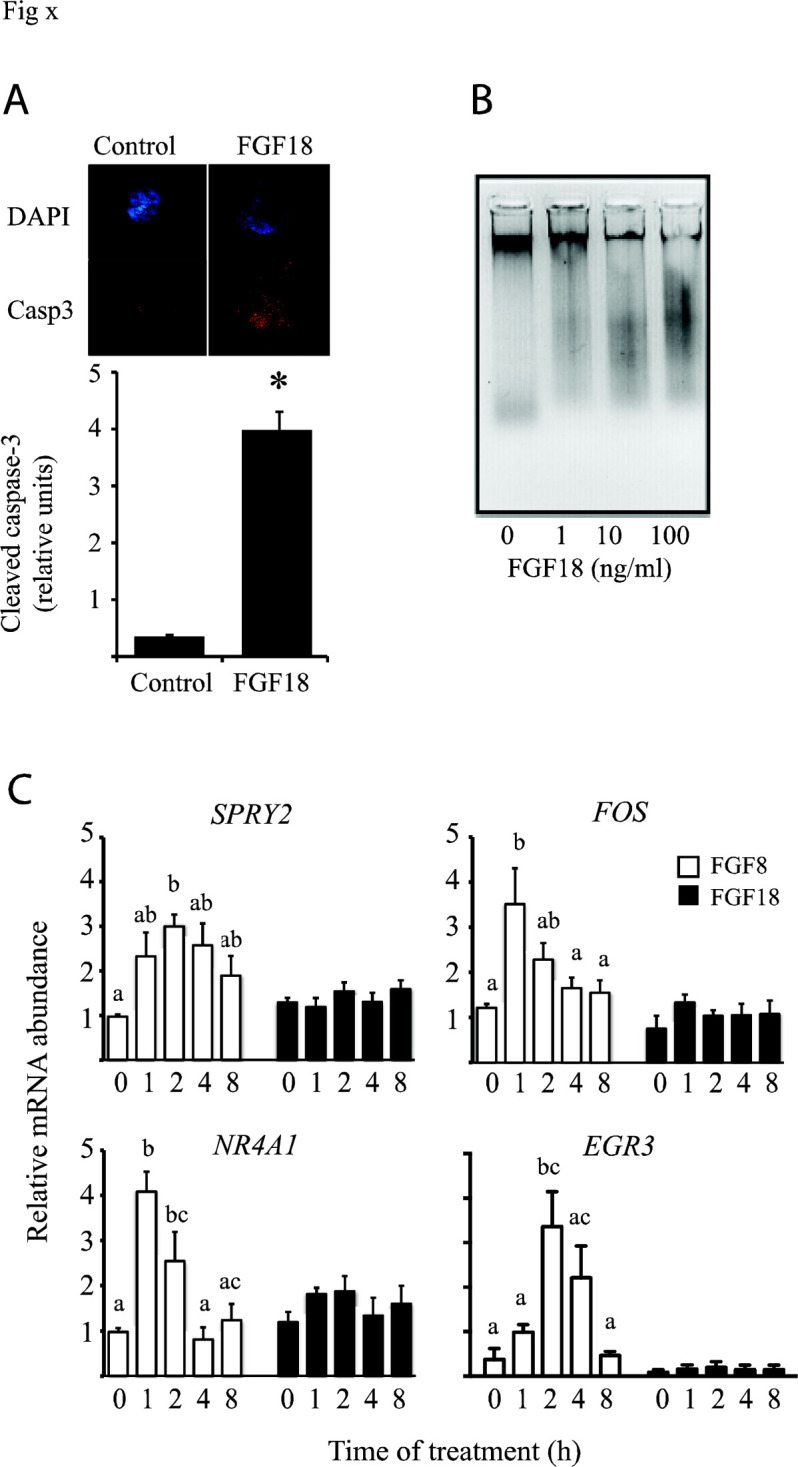
FGF18 increases apoptosis in granulosa cells and does not activate typical FGF signaling pathways. Culture of bovine granulosa cells with recombinant human FGF18 increases cleaved caspase-3 protein levels (A) and DNA fragmentation (B), and addition of FGF18 (10 ng/ml, filled bars) does not increase levels of mRNA of typical response genes (compare with FGF8; 10 ng/ml, hollow bars). Bars with common letters are not statistically different. Data from (Portela *et al*., 2010, 2015; Jiang *et al*., 2013; Han *et al*., 2017).

FGF18 has been clearly demonstrated as a pro- apoptotic factor. This member of the fibroblast growth factor family is produced in vivo by the theca layer in cattle, and protein and mRNA levels are higher in atretic compared with healthy follicles. Moreover, recombinant FGF18 inhibits granulosa cell estradiol secretion and abundance of *CYP19A1, CYP11A1, HSD17β1, STAR, HSD3β1* and *FSHR* mRNA (Portela *et al*., 2010), and increases DNA fragmentation and abundance of cleaved caspase-3 in granulosa cells (Portela *et al*., 2010, 2015; Fig. 1). Injecting FGF18 into the growing dominant follicle *in vivo* caused follicle regression (Portela *et al*., 2015). It is interesting to note here that some growth factors promote granulosa cell proliferation but decrease estradiol secretion in vitro, FGF9 for example (Schreiber and Spicer, 2012); this apparent paradox has been referred to a dedifferentiating effect, but proliferation of cells may be caused by growth factor activation of MAPK pathways that drive proliferation irrespective of lower estradiol levels.

In support of this notion, FGF18 appears not to activate the typical FGF signalling pathways in granulosa cells; specifically, FGF18 does not increase MAPK3/1 phosphorylation or abundance of typical FGF response genes including SPRY2 and EGR3 (Jiang *et al*., 2013; Han *et al*., 2017), although it does increase MAPK14 phosphorylation (Portela *et al.,* 2015). The mechanism of action of FGF18 remains obscure.

### The transforming growth factor-β (TGFβ) family

**Figure 2:**
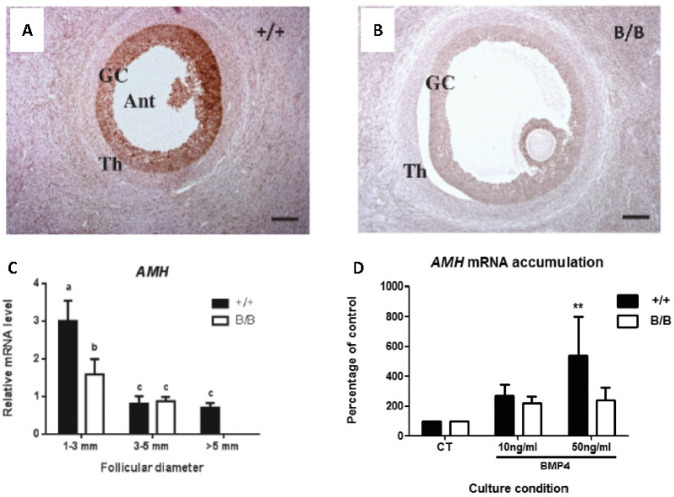
Regulation of AMH by BMPs in sheep follicles. Anti-Mullerian hormone protein (A,B) and mRNA (C) levels are markedly reduced in sheep carrying the hyperprolificacy Booroola mutation in BMPR1 compared to non- carriers, and this mutation leads to reduced granulosa cell responsiveness to BMP4 (D). Bars with common letters are not statistically different, and asterisks denote a significant stimulation of *AMH* mRNA abundance by BMP4. Data from Estienne *et al*. (2015).

Anti-Müllerian Hormone (AMH), another member of the TGFβ super-family, is secreted by granulosa cells of small follicles and is known to inhibit the recruitment of primordial follicles in rodents (Durlinger *et al*., 1999) but not in sheep (Campbell *et al*., 2012). In ruminants, as in non-ruminant species, AMH levels decrease with increasing size of antral follicles, and appears to be inversely correlated with CYP19A1 expression (Monniaux *et al*., 2008; Campbell *et al*., 2012; Liang *et al*., 2016). Recombinant AMH decreases granulosa and theca cell steroidogenesis in vitro (Campbell *et al*., 2012), and has been shown to increase apoptosis in human granulosa tumour cells (Anttonen *et al*., 2011). AMH mRNA levels and protein secretion from granulosa cells in vitro are stimulated by BMP2, BMP4 and BMP6 in sheep and cattle (Rico *et al*., 2011; Estienne *et al*., 2015), and *AMH* mRNA levels are increased by BMP15 in sheep granulosa cells, and GDF9 enhanced this effect (Pierre *et al*., 2016). At least part of the ability of the *FecB* mutation to decrease granulosa apoptosis may be the reduced expression of AMH mRNA and protein in this genotype (Fig. 2).

**Figure 3:**
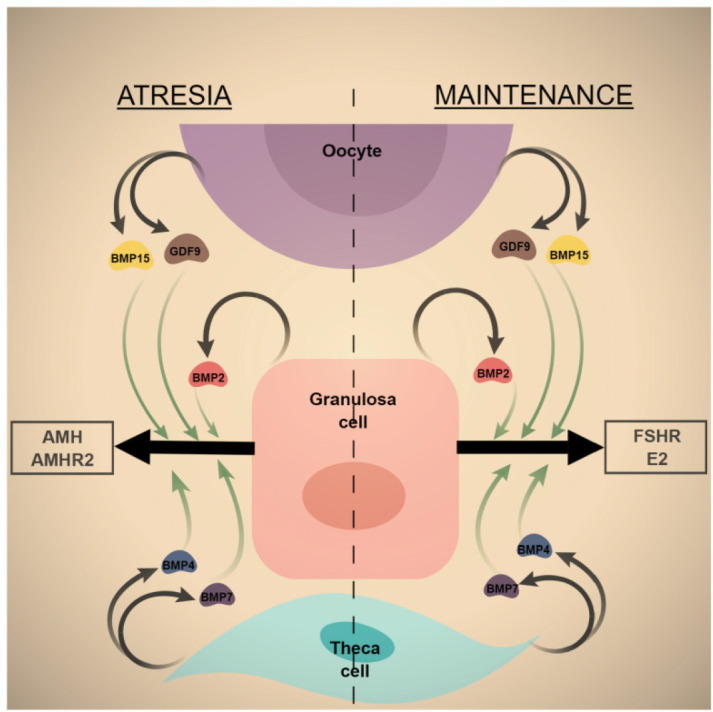
The duality of BMP action in the ovarian follicle. BMPs of theca, oocyte and granulosa cell origin have been shown to stimulate FSHR expression and estradiol secretion from granulosa cells, thus supporting granulosa cell survival and growth or maintenance of the dominant follicle. However, these same factors have also been shown to increase AMHR2 expression and AMH secretion, which is a pro-apoptotic signal. The net impact on the follicle is likely determined by other endocrine/paracrine factors present during the static phase of the dominant follicle lifespan.

It is worthy of mention that certain BMP family members appear to have both pro-survival and pro- apoptotic actions, as they have been shown to stimulate estradiol secretion, which is a pro-survival factor, or stimulate AMH secretion which promotes apoptosis (Fig. 3). It is most likely that the predominant activity depends on stage of follicle growth and endocrine/paracrine milieu of the follicle at the time in question. Studies in which multiple endocrine/paracrine factors are studied in combination are needed to resolve this enigma.

### miRNA

The roles of miRNA in dominant follicle (Fan *et al*.,2016) and miR-142 in cancer cells (Li *et al*., 2016). However, miR-150 promotes cell growth in ovarian cancer (Li *et al*., 2015) but causes apoptosis in development remain obscure. Some miRNA upregulated in atretic follicles (Table 1) have been shown to block apoptosis, including miR-21 in mouse granulosa cells (Carletti *et al*., 2010), miR-149 in lymphoma cells lymphocytes (Sang *et al*., 2016) as well as endothelial cells. It is possible that these miRNAs are upregulated in atretic follicles as a defence against apoptosis, and are thus an effect of atresia rather than a cause.

## Conclusions

As growth of the dominant follicle slows, the follicle faces two possible fates: maintenance of growth/survival, and atresia. Increases in gonadotropin concentrations will drive survival, likely through increased intrafollicular estradiol, IGF1 and mRNA levels, and evidence is accumulating that other intrafollicular factors may either predispose the follicle to or provide protection against atresia. The pro- apoptotic factors likely include increased secretion of

**Figure 4:**
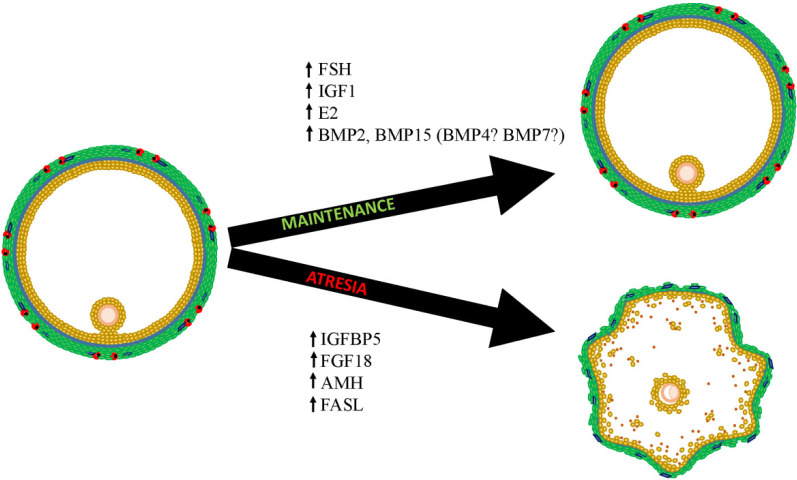
Schematic representation of the fate of the dominant follicle as it enters the static phase of its lifespan. The follicle may survive and go on to the preovulatory stage if appropriate gonadotrophic stimuli are provided. Alternatively, lack of gonadotropin support in combination with the secretion of local proapoptotic factors including but not limited to FGF18 and AMH may initiate FASLG-mediated granulosa cell apoptosis and irrevocably drive the follicle into regression.

AMH by granulosa cells and of FGF18 by theca cells, which increase FASLG-mediated apoptosis of granulosa cells and thus initiate an irreversible process of follicle atresia. The BMPs have been implicated, and they may help or hinder follicle survival depending perhaps on other endocrine or paracrine factors present. The potential role of each is summarized in Fig 4. Exactly when and how this fate determination occurs is unknown, and future research is required to determine the paracrine and autocrine events that occur within the follicle during the static phase of its lifespan.
